# Beyond the scope of the HPV triage strategy – proposals for consideration

**DOI:** 10.1111/1471-0528.15983

**Published:** 2019-11-05

**Authors:** B Böttcher, S Abdel Azim

**Affiliations:** ^1^ Department of Gynaecological Endocrinology and Reproductive Medicine Medical University of Innsbruck Innsbruck Austria; ^2^ Department of Gynaecology and Obstetrics Medical University of Innsbruck Innsbruck Austria

This sub‐analysis from the ARTISTIC trial cohort aims to identify women at high risk for CIN3 lesions in order to avoid overtreatment and to develop a cost‐effective triage strategy (Gilham et al. *BJOG* 2020;127:58–68). The authors investigated whether HPV‐positive women with normal cytology or low‐grade lesions could be screened less often, with women who are HPV16/18 being screened annually and those women with other high‐risk HPV infections being screened every 2 years. With this strategy, a minimal increased incidence of microinvasive cancer has to be accepted, with the cost of its subsequent treatment justified before it can be implemented. To address this issue, a cost–benefit analysis should be undertaken by health economists using the data in this study.

According to the authors, this extended screening period triage strategy may not be applicable to women older than the age of 45 years, as the risk of invasive cervical cancer was higher in this age group than in women ≤45 years old. The authors suggested that post‐menopausal women with persistent HPV infection may opt to have immediate colposcopy and possible therapeutic excision instead. However, the cut‐off age of 45 years does not reflect the mean age of menopause from an endocrinological perspective. Before implementing this new triage strategy, it should be decided whether age >45 years old or menopausal status is used as the criterion for advising for repeated screening or immediate cervical loop excision, or whether this decision should made on an individual basis.

In some guidelines, it is recommended to start HPV screening at the age of 30 years (Wright et al. *Obstet Gynecol* 2004;103:304–9; Meijer et al. *Int J Cancer* 2009;124:516–20). At the time of the ARTISTIC trial, screening was initiated at the age of 20. Currently, screening for cervical cancer in the UK starts at the age of 25 and, according to the authors, there is little difference in the incidence of CIN3 detected in women in the age groups 20–24 and 25–29 years.

Furthermore, the psychological impact of the proposed change to the screening triage strategy is beyond the scope of this paper. We do not know whether extended screening periods of 2 years will be well accepted by HPV‐positive women. Some women might require earlier reassurance that no progression to a microinvasive carcinoma has occurred. Evidence is scarce, and it has been shown previously that women suffer from negative emotions such as fear and anxiety before their first cervical screening or colposcopy, even after the implementation of HPV vaccination (Young et al. *BMC Womens Health* 2018;18:200). On the other hand, the data may help to reassure women about the low risk of CIN3 after resolution of their HPV infection. This will be an area for future research

## Disclosure of interests

No disclosures. Completed disclosure of interest forms are available to view online as supporting information.
